# Two years into COVID‐19 – Lessons in SARS‐CoV‐2 and a perspective from papers in *FEBS Letters*

**DOI:** 10.1002/1873-3468.14226

**Published:** 2021-11-16

**Authors:** Urs F. Greber

**Affiliations:** ^1^ Department of Molecular Life Sciences University of Zürich Switzerland

## Abstract

The 2019 outbreak of coronavirus disease (COVID‐19) in Wuhan (Hubei province of China) has given rise to a pandemic spread of virus, more than 240 million incidences and a death toll larger than 5 million people. COVID‐19 has set off large efforts in research, therapy and patient care, as well as public and private debates in every imaginable form. A number of scientists used the publication platforms provided by the Federation of the European Biochemical Societies (FEBS) to present their research data, reviews, opinions and other contributions relating to COVID‐19 and severe acute respiratory syndrome coronavirus 2 (SARS‐CoV‐2). Here, I highlight the recent COVID‐19 papers which have been published and collected in a Virtual Issue in *FEBS Letters*, and discuss their implications towards understanding the molecular, biochemical and cellular mechanisms of SARS‐CoV‐2 infections, vaccine development and antiviral discovery strategies.

## Introduction

In December 2019, a local outbreak of pneumonia caused by a previously unknown coronavirus was reported in Wuhan (Hubei, China) for early discussions in *Febs Letters*, see [1, 2, 3]. Severe acute respiratory syndrome coronavirus 2 and its variants have caused a significant pandemic with several hundred millions of reported cases and nearly 5 million deaths across the world, as of October 2021 (https://coronavirus.jhu.edu/map.html). Coronaviruses had reached centre stage in 2002/03 when SARS‐CoV (of bat and palm civet origin) caused a limited epidemic outbreak, and Middle East respiratory syndrome (MERS) coronavirus (from camels) was first detected to cause severe human respiratory disease on the Arabian Peninsula in 2012.

CoVs are enveloped viruses of the *Coronavirinae* subfamily with a positive (+) sense single‐stranded RNA genome. Based on their genomic and phylogenetic structures, the *Coronavirinae* comprise four genera, α‐ β‐, γ‐ and δ‐CoV [4]. This genus nomenclature is not to be confused with the nomenclature for variants of concern (VoC) recently introduced by the World Health Organization (WHO). CoVs cause mild to severe infections of human and animal respiratory tracts, often with cold‐like symptoms, exemplified by the four endemic human CoVs, 229E (α‐CoV, reservoirs in bats), NL63 (α‐CoV, reservoirs in bats), OC43 (β‐CoV, reservoirs in domesticated animals) and HKU1 (β‐CoV, reservoir in mice) [5]. SARS‐CoV‐2 (β‐CoV) may become (or is already considered) the fifth human endemic CoV, as it continues to infect humans [6]. It likely originated from bats in South‐East Asia through a yet unknown intermediary host(s) [7, 8]. Unlike SARS‐CoV in 2002/03, SARS‐CoV‐2 is highly capable of human‐to‐human transmission, causes frequent asymptomatic infections and disseminates through superspreader events. Understanding the molecular mechanisms of CoV infections is crucial to tackle not only how the virus infects, spreads and deregulates the immune system, but also how it persists in infected cells for extended periods of time. The latter is thought to be important for genetic recombination and furthering viral divergence. This FEBS Letters collection of SARS‐CoV‐2 papers addresses fundamental research questions, drug discovery and vaccination strategies.

## Infection mechanisms – receptors, trafficking, replication

All viruses require cell surface receptors for infection, but not all viruses in a given family use the same receptor, and often a particular virus uses different receptors depending on the cell type. Virus–receptor interactions lead to infection or virus inactivation [9, 10]. Surface receptors provide direct contacts for the virion with the plasma membrane and trigger virion uptake or at least uptake of the viral genome into the cell. The former occurs by receptor‐mediated endocytosis and the latter upon fusion of the viral envelope with the plasma membrane [11]. Attachment factors are distinct from receptors, as they bind to the virion but do not lead to infection or clearance [9]. Receptors and attachment factors are contrasted by a third class of cell surface molecules, facilitators, which support the function of receptors (for further discussion, see [9]). These distinctions are important, as scientists are increasingly interested in understanding the mechanisms of cell‐to‐cell variability of virus infections, which is in part dictated by virus binding to cells, and the abundance of receptors, attachment factors and facilitators [12, 13, 14].

The early evolution of SARS‐CoV‐2 in humans gave rise to a particular point mutation in the receptor‐binding domain (RBD) of the viral spike (S)‐protein, a substitution of asparagine to tyrosine at position 501 (N501Y). N501Y is found in several VoC, including the alpha, beta and gamma variants. Luan *et al*. used all‐atom molecular dynamics simulations to explore why the N501Y S‐protein binds better to the hACE2 receptor than the original S‐protein [15]. Improved binding of N501Y was predicted to be due to Y501 π–π and π–cation interactions with Y41 and K353 of hACE2, respectively. Gratifyingly, this notion was then confirmed by direct binding assays including surface plasmon resonance and atomic force microscopy [16]. Given that the highly prevalent delta VoC lacks the N501Y mutation, the results raise the question if S‐protein has evolved the maximal strength of binding to hACE2, or if the S‐protein can evolve additional features to enhance productive binding of SARS‐CoV‐2 to hACE2.

The extracellular domain of S has another intriguing feature, a polybasic furin cleavage site, which has been subject to much discussion and speculation. While the MERS S‐protein also harbours a furin cleavage site, the closest relatives of SARS‐CoV‐2, bat β‐CoVs RaTG13 or BANAL‐52 lack this feature (see https://doi.org/10.21203/rs.3.rs‐871965/v1). Furin cleavage occurs during S‐protein biogenesis in acidic compartments of the secretory pathway and enables S‐protein binding to neuropilin 1 via the C terminus of the cleaved S1 domain enhancing infection of epithelial and olfactory neuronal cells low in transmembrane protease serine subtype 2 (TMPRSS2) [17, 18, 19]. Intriguingly, the S1/S2 cleavage site has been maintained in human evolution of SARS‐CoV‐2, suggesting that it provides a fitness advantage for the virus. On the cell surface, TMPRSS2 cleaves the furin‐activated S‐protein at the proximal S2’ site. In absence of TMPRSS2 cleavage, the membrane‐bound S2 protein containing the fusion peptide of the incoming virus particle is proteolysed by cathepsin L in acidic endosomes enhancing membrane fusion and infection (reviewed in [20]). Jennings *et al*. explored the intracellular trafficking of the S‐protein and found that the cytoplasmic tail of S contains a coatomer protein I binding motif, which enables S‐protein recycling within the Golgi apparatus, facilitates S‐protein cleavage and glycosylation, and localization of functional S on the plasma membrane of infected cells [21]. This is key for S‐protein to mediate cell–cell fusion, spreading of subviral complexes including the viral RNA genome, as well as infectivity of the progeny virions.

Akin to many other viruses, SARS‐CoV‐2 binds to multiple cell surface molecules, including receptors and attachment factors, such as heparan sulphate (HS), a sulphated glycosaminoglycan (GAG). Watanabe and colleagues developed highly sensitive fluorescence‐based GAG microarrays and identified the binding of SARS‐CoV‐2 S‐protein to chondroitin sulphate E (CSE) and confirmed the binding to HS [22]. Intriguingly, S1 bound to heparin and S2 to both CSE and HS (as well as heparin). It will be interesting to explore the biological significance of these interactions.

Any productive virus infection requires the replication of the viral genome. While persistent infections make just a few copies, lytic infections yield hundreds or thousands of copies per cell [23, 24]. Viral (+) sense RNA genomes can be readily translated and replicated on cytoplasmic organelles, such as ER‐ or Golgi‐derived membranes [25, 26]. For example, the replication membranes grow over time in lytic infections and require the steady supply of lipids, including phospholipids, such as phosphatidyl‐inositol or phosphatidyl‐choline, and cholesterol furnished by lipid exchange proteins at membrane contact sites between different organelles [for an example see enteroviruses, [25, 27]]. Enterovirus replication critically requires the exchange protein oxysterol‐binding protein (OSBP) 1, which docks to an ER protein, the vesicle‐associated membrane protein‐associated protein (VAP)‐A by virtue of its FFAT motif, and to the Golgi membrane by its PH domain binding to PI4‐phosphate (PI4P) [28, 29]. OSBP1 shuttles cholesterol and PI4P between ER and Golgi at membrane contact sites and drives a counter‐current lipid flux for membrane growth. Using in‐solution NMR, Furuita and colleagues identified an FFAT‐like motif in the SARS‐CoV‐2 RNA‐dependent RNA polymerase (RdRP), which specifically binds to the major sperm protein domain of VAP‐A [30]. Such mechanism might ensure appropriate localization of RdRP on the viral replication membrane. Whether the native RdRP binds to VAP and how VAP binding contributes to viral replication awaits future investigations.

## Antiviral compounds targeting the virus

Arguably, antiviral drug development is a top priority in the management of COVID‐19. It has received many‐fold contributions from academic as well as small and medium enterprises, and increasingly also big pharma. Two principal approaches to identify antiviral drugs are feasible. One is repurposing existing chemical compounds approved for human use outside of COVID‐19 [see, e.g., 31, 32], and another one is *de novo* drug development, including biologicals, but also small chemical compounds against viral enzymes, such as the viral RNA polymerase and its two proteases (nsp3 PL^pro^ and nsp5 M^pro^) cleaving the viral polyprotein, and also host proteins, including ubiquitin thereby blunting antiviral host defence. Hijikata *et al*. constructed structural atomic models of SARS‐CoV‐2 proteins in complex with drug‐like template molecules and compared the results with approved or experimental compounds, including natural medicines [33]. Results suggested several molecules for further studies against SARS‐CoV‐2, including carfilzomib, sinefungin, tecadenoson and trabodenoson.

Padhi and colleagues used computational methods to model the favipiravir‐binding site in SARS‐CoV‐2 RdRP and thereby uncovered potential mutations conferring drug resistance [34]. Favipiravir is an antiviral drug approved in Japan to treat pandemic influenza virus infections. The phosphorylated prodrug is incorporated by viral RdRP enzymes and functions as an RNA mutagen [35]. Upon pretreatment, favipiravir showed antiviral efficacy against SARS‐CoV‐2 in hamsters [36]. Favipiravir is in clinical trials against mild and intermediate forms of COVID‐19 [37]. Padhi *et al*. focussed on chain‐termination induced by favipiravir. They used a high‐throughput interface‐based protein design to generate more than 100’000 variations of the favipiravir‐binding site on the RdRP. They identified mutational hot spots and showed that just a few mutations in RdRP suffice to confer favipiravir resistance. Strikingly, the calculations also implied that dozens of drug resistance mutations exist in SARS‐CoV‐2 genomes in the CoV‐GLUE database. This implies that drug‐resistant viruses circulate in humans, even in the absence of significant drug selection pressure.

For direct antivirals to be effective against SARS‐CoV‐2 on the long run, multiple antiviral treatments are likely required, akin to the highly successful combo‐therapy, essentially curing hepatitis C virus infections [38]. One of these targets is the virus interaction with the ACE2 receptor. To identify an allosteric druggable site within the RBD of the SARS‐CoV‐2 S‐protein, Bhattacharjee *et al*. used molecular dynamics simulations of the apo‐RBD and the ACE2 receptor‐bound RBD and could reveal how correlated motions and electrostatic energy are part of the allosteric crosstalk in the RBD upon receptor binding [39]. In particular, electrostatic energy perturbations appeared to determine favourable pairwise crosstalk within the RBD residues upon binding to ACE2. It was interesting to note that the allosteric path in the RBD comprises evolutionarily conserved residues present in closely related coronaviruses, suggesting that targeting these residues with chemical agents may have potential for antiviral treatment. On the short run, direct topical antiviral therapies may also be considered, as sketched out in a conceptual paper by Honarmand Ebrahimi, who proposed to engineer upper respiratory tract bacteria, such that they secrete SARS‐CoV‐2 S‐protein binding proteins, neutralize virus and thereby reduce infection [40].

## Host‐directed antivirals

Identifying and developing antivirals has been challenging for SARS‐CoV‐2 and most other viruses for two main reasons. One is that direct antiviral compounds give rise to drug‐resistant mutants, and two that severe COVID cases not only exhibit high viral load but also strongly dysregulate the inflammatory response, and cause cytokine storms [41]. The latter includes transforming growth factor (TGF) beta 1, possibly depending on the TGF‐beta1 activator THBS3, which is genetically associated with severe COVID‐19 cases, as suggested by the COVID‐19 Host Genetics Initiative (https://www.covid19hg.org). This topic is highlighted in the study by Wang *et al*. [42]. Whether it relates to the increased COVID‐19 risk in patients suffering from inheritable mucopolysaccharidosis (MPS) and thickened mucus in the lungs is unknown. Intriguingly, however, results from Pierzynowska *et al*. obtained with MPS cell lines suggest that several genes involved in SARS‐CoV‐2 biogenesis and antiviral defence are expressed at reduced and increased levels, respectively, compared to non‐MPS cells, suggesting that these cells are less susceptible to SARS‐CoV‐2 than control cells [43]. To understand these observations, mechanistic studies are now required.

An interesting conceptual contribution was provided by Yaneske and colleagues who developed genome‐wide metabolic modelling based on pre‐existing transcriptomic and proteomic data to show that SARS‐CoV‐2 infection of cancer cells goes along with a metabolic shift to glycolytic energy production [44]. The work predicts intriguing roles of translation, ribosomal RNA metabolism and fatty acid biosynthesis in rewiring cytokine secretion, and energy production pathways. Targeting cell metabolism, and in particular fatty acid synthesis, appears to be a particularly interesting strategy to explore broadly acting antiviral treatments beyond coronaviruses, as shown by ground‐breaking work with cytomegalovirus a number of years ago [45]. In fact, inhibition of fatty acid synthase by highly specific chemicals was shown to be effective against respiratory syncytial virus, human parainfluenza and rhinovirus in proof‐of‐concept studies [46, 47].

The concept of targeting the host rather than the virus may have several advantages, including a much broader range of antiviral effects and higher viral evolutionary cost for evasion of the antiviral action. The latter was recently demonstrated with rhinovirus (common cold causing agent), which adapts to endosomal acidification inhibitors, such as the antiparasitic salicylanilide niclosamide, by evolving critical capsid mutations in its interprotomeric interface [48]. These mutations destabilize the virion and reduce viral fitness. They are necessary (as shown by reproducible evolution experiments) and sufficient (shown by reverse genetics) for the virus to infect cells lacking acidic endosomal pH, which is a critical uncoating cue for wild‐type infections. These results support host‐directed antiviral treatments. Of note, niclosamide is used to treat, for example, human tapeworm infections [49] and is a candidate for inhalation treatment of COVID‐19 [50].

## COVID vaccines and mRNA

As of today, the most effective measure against COVID‐19 is vaccination. Immunization by vaccination drives the immune system to generate cellular and antibody‐based responses against the antigen of interest, while passive immunization delivers therapeutic antibodies directly into the body [51]. The latter may be of benefit to individuals in need of acute treatment, but broad applications are limited by availability and high cost.

The most widely used vaccines against COVID‐19 are virus vectors as well as mRNA‐based therapies. The former is mostly driven by adenovirus‐based vectors delivering the gene encoding the S‐protein to the nucleus for transcription, and cytoplasmic protein synthesis and surface antigen presentation to immune cells [52], while the latter is based on injection of mRNA encoding the S‐protein, bypassing the nuclear step [53, 54]. One of the early proponents of the mRNA technology, Drew Weissman, has shared his personal insights into mRNA vaccines in an interview with Daniela Ruffell [55]. An important early observation was that chemical RNA modification attenuates the activation of dendritic cells. The key modification turned out to be the replacement of uridine residues with pseudouridine (Psi), the most common RNA modification, for example occurring in nonimmunogenic RNAs, such as transfer RNAs or ribosomal RNAs [56]. At the molecular level, Lin and colleagues showed that Psi residues establish additional bonds with neighbouring atoms and thereby strengthen RNA–RNA and RNA–protein interactions [57]. In case of messenger RNA, the presence of Psi sites enhances the lifetime and the translation efficiency of the mRNA and reduces unwanted innate immune reactions, such as inflammation.

One limitation of the currently approved SARS‐CoV‐2 vaccines has been that multiple doses were required for optimal protection against COVID‐19. Salzer and colleagues used a ferritin‐like iron binding protein as a scaffold to chemically couple the SARS‐CoV‐2 RBD and thereby provide stable multivalent dodecameric vaccine nanoparticles [58]. The investigators could show that single‐dose immunization with a multimerized SARS‐CoV‐2 receptor‐binding domain (RBD) induced a higher antibody titre and enhanced neutralizing antibody response compared to monomeric RBD. It protected hACE2‐expressing mice from serious illness and led to viral clearance from the lungs upon SARS‐CoV‐2 infection. This argues that multimerization of SARS‐CoV‐2 subunits on a highly stable scaffold provides an interesting protein‐based vaccine platform against COVID‐19.

## Conclusion

Considering the large diversity of coronaviruses and their zoonotic and anthroponotic transmissions [4], the prediction is that these viruses (besides other viruses) will continue to be significant threats to human health. From a scientific point of view, it makes sense to enhance virus surveillance efforts at the interface between humans and animals and develop innovative strategies for studying thus far unknown aspects of viruses and their intricate interactions with hosts. All this will be helpful to reduce the threats from SARS‐CoV‐2 VoC, as well as viruses emerging in the future. The scientific community is well advised to work together in all disciplines and share information as effectively as in the current situation. I have no doubt that this strategy is not only doable, but will contribute to increase resilience of societies and respectful human behaviour.
